# Changes in the lipopolysaccharide of *Proteus mirabilis* 9B-m (O11a) clinical strain in response to planktonic or biofilm type of growth

**DOI:** 10.1007/s00430-018-0534-5

**Published:** 2018-01-12

**Authors:** Agnieszka Zabłotni, Dominik Matusiak, Nikolay P. Arbatsky, Magdalena Moryl, Anna Maciejewska, Anna N. Kondakova, Alexander S. Shashkov, Czesław Ługowski, Yuriy A. Knirel, Antoni Różalski

**Affiliations:** 1 Laboratory of General Microbiology, Department of Biology of Bacteria, Institute of Microbiology, Biotechnology and Immunology, University of Łódź, Banacha 12/16, 90-237 Łódź, Poland; 2 Department of Biology of Bacteria, Institute of Microbiology, Biotechnology and Immunology, University of Łódź, Banacha 12/16, 90-237 Łódź, Poland; 3 Department of Immunochemistry, Hirszfeld Institute of Immunology and Experimental Therapy, Polish Academy of Sciences, Rudolf Weigl 12, 53-114 Wrocław, Poland; 4 N. D. Zelinsky Institute of Organic Chemistry, Russian Academy of Sciences, Leninsky Prospekt 47, 119991 Moscow, Russia

**Keywords:** *Proteus*, Biofilm, Planktonic form, Lipopolysaccharide, Core region

## Abstract

The impact of planktonic and biofilm lifestyles of the clinical isolate *Proteus mirabilis* 9B-m on its lipopolysaccharide (O-polysaccharide, core region, and lipid A) was evaluated. *Proteus mirabilis* bacteria are able to form biofilm and lipopolysaccharide is one of the factors involved in the biofilm formation. Lipopolysaccharide was isolated from planktonic and biofilm cells of the investigated strain and analyzed by SDS–PAGE with silver staining, Western blotting and ELISA, as well as NMR and matrix-assisted laser desorption ionization time-of-flight mass spectrometry techniques. Chemical and NMR spectroscopic analyses revealed that the structure of the O-polysaccharide of *P. mirabilis* 9B-m strain did not depend on the form of cell growth, but the full-length chains of the O-antigen were reduced when bacteria grew in biofilm. The study also revealed structural modifications of the core region in the lipopolysaccharide of biofilm-associated cells—peaks assigned to compounds absent in cells from the planktonic culture and not previously detected in any of the known *Proteus* core oligosaccharides. No differences in the lipid A structure were observed. In summary, our study demonstrated for the first time that changes in the lifestyle of *P. mirabilis* bacteria leads to the modifications of their important virulence factor—lipopolysaccharide.

## Introduction

Bacteria belonging to the genus *Proteus* are rod-shaped, Gram-negative, nonsporulating microorganisms which, due to their biochemical properties, are widely distributed in environment [1, 2]. The systematics of these bacteria have been changed several times, since they were first described by Hauser in 1885, and currently, the genus is divided into the following species: *P. mirabilis, P. vulgaris, P. penneri, P. hauseri, P. myxofaciens, P. terrae* sp. nov., and *P. cibarius* sp. nov. as well as three unnamed *Proteus* genomospecies: 4, 5, and 6 [1, 3–6]. Except for *P. myxofaciens, P. terrae* sp. nov., and *P. cibarius* sp. nov., the strains from the mentioned species are classified as opportunistic pathogens. They are responsible for various human diseases such as meningitis, pneumonia, wounds and bone infections, brain abscesses, and bacteremia [7–9]. The most frequent among *Proteus-*mediated infections are recurrent urinary tract infections (UTIs), whose hallmark is formation of urinary stones, containing struvite and carbonate apatite. *P. mirabilis* strains are mentioned as the main etiological factor of these infections. The frequency of *P. mirabilis* infections rises during catheterization, especially in long-term catheterized patients [3, 10, 11]. The recurrent character and formation of urinary stones are the main problems in combating *P. mirabilis* infections, but bacterial biofilm, especially that formed on the catheter surface, may also complicate the treatment [12]. A unique feature of biofilms developed by *Proteus* rods is the fact that they are covered with struvite and apatite crystals, which precipitate in urine. Such biofilms are less sensitive to antimicrobial drugs, as well as more resistant to the host immune system [13, 14].

Biofilm is a type of growth in which bacteria can attach to a solid surface and develop a well-organised structure. This form of bacterial population is immersed in self-produced, extracellular polymeric substances, called matrix. Many various factors are involved in the formation of a complex biofilm structure. One of these factors is lipopolysaccharide (LPS)—a component of the outer membrane of Gram-negative bacteria. LPS consists of three regions: the most external O-polysaccharide (OPS), the core region, and lipid A. LPS possessing all three regions is characteristic for smooth forms of bacteria and is not only responsible for the emergence of a wide spectrum of biological reactions in the cells of macroorganisms (especially endotoxic activity connected with the lipid A part), but also plays a role in the interactions between the bacterial cells and cells of an infected host [15–17]. Due to their acidic character, O-polysaccharide chains of *Proteus* LPS contribute to the urinary stones occurrence, and also participate in biofilm matrix formation [1, 18]. This extracellular matrix enhances the adherence of *Proteus* rods to the solid surface and biofilm development.

It has been well demonstrated that sessile bacteria differ greatly from their planktonic counterparts, especially with regard to drug resistance. Many bacteria living in biofilm exhibit reduced (from 10 up to 1000 times) susceptibility to a broad range of antimicrobial agents in comparison with their planktonic forms [19, 20]. Benamara and co-workers [21] showed that in *Pseudomonas aeruginosa* sessile cells, the inner membrane lipidome shows a decrease in the amount of the uneven-numbered chain phospholipids and accumulation of phosphatidylethanolamines with long-chain lipids. Little is known about changes in the structure of lipopolysaccharides (LPSs) and their properties depending on the form of bacterial growth; however, also in *P. aeruginosa*, it has been demonstrated that the chemical structure of this component, essential for the outer membrane stability, can be modified [22]. The lack of data concerning possible modifications of *Proteus* bacteria LPSs in different forms of growth encouraged us to investigate this problem. Therefore, the aim of this study was to check whether a form of growth (planktonic or biofilm) of the investigated *P. mirabilis* strain leads to structural modifications of its LPS, changes in the serological reactions profile or particular biological properties.

## Materials and methods

### Bacterial strain, antiserum, culture conditions, extraction of lipopolysaccharides

The *Proteus mirabilis* 9B-m (O11a) strain was isolated from urine of a patient of Barlicki Hospital in Łódź (Poland), and was kindly provided by D. Drzewiecka, Ph.D., the Laboratory of General Microbiology, University of Łódź. The strain was selected for the study on the basis of the results of preliminary investigation (data not shown) which demonstrated its great ability to form biofilm, in flat-bottomed polystyrene plates, among 30 tested clinical *Proteus* spp. isolates. Bacteria were stored in Luria broth with 25% glycerol, at − 80 °C. The polyclonal rabbit serum against the investigated *P. mirabilis* 9B-m strain (obtained as described by Sidorczyk and co-workers [23]) came from the Laboratory of General Microbiology, University of Łódź, Poland.

Planktonic as well as biofilm-associated bacteria were cultivated in nutrient broth (BTL, Poland). Biomass of planktonic cells was obtained by aeration of the bacteria in liquid medium at 37 °C for 18 h. Next, the bacteria were killed by the addition of phenol at 1% concentration, washed twice with distilled water, centrifuged, and finally lyophilized.

To obtain biomass of sessile bacteria, the microorganisms were cultured at 37 °C for 72 h in a sterile glass bioreactor with a constant medium flow (1 ml per minute), fitted with vertical glass plates for biofilm formation. Obtained biofilm was collected manually, washed twice with water and centrifuged. Next, the bacteria were inactivated by moist heat sterilization (115 °C, 20 min) and lyophilized.

To demonstrate that after changing the lifestyle from biofilm to the planktonic form the investigated strain regain its ability to synthesize full-length-LPS, sessile bacteria were transferred to liquid medium and cultured in planktonic conditions described above.

LPSs from dry masses of planktonic and biofilm-forming bacteria were extracted using the phenol–water method by Westphal and Jann [24], modified as described by Palusiak [25]. They were subsequently used for silver staining, stimulation of THP-1 cells, and as antigens in serological assays, as well as for the chemical analysis of O-polysaccharides, core regions, and lipids A.

### Silver staining of lipopolysaccharides and serological assays

The LPSs (3 µg) from both types of cultures were examined by sodium dodecyl sulfate–polyacrylamide gel electrophoresis (SDS–PAGE) with further Alcian blue prestaining [26] and subsequent silver staining of separated samples according to Tsai and Frasch [27].

Serological investigations were performed using the ELISA and the Western-blotting technique described in detail by Sidorczyk et al. [23]. For the Western blotting, 2 µg (short path) and 5 µg (extended path of separation) of each LPS in the loading buffer were added per single lane. In ELISA, native as well as adsorbed polyclonal O-antisera against *P. mirabilis* 9B-m were used. Adsorption of the antibodies from the serum was carried out by a threefold addition of whole cells of the planktonic or sessile bacteria to the diluted serum according to the method given by Drzewiecka et al. [28].

### THP-1 cell culture, differentiation, and lipopolysaccharide stimulation

THP-1 cells (ATCC TIB-2) were cultured in RPMI 1640 medium, supplemented with 10% heat-inactivated fetal calf serum, 2 mM L-glutamine, 100 U penicillin ml^−1^, and 100 µg ml^−1^ streptomycin (all components obtained from Cytogen). The culture was performed at 37 °C, in a moist atmosphere containing 5% CO_2_. THP-1 cells in RPMI medium (at a density of 10^5^ ml^−1^) were differentiated into a macrophage-like phenotype by the treatment with PMA (Phorbol Myristate Acetate); (50 nM, Sigma) for 48 h. Next, the cells were stimulated with LPSs suspended in a fresh medium, obtained from biofilm and planktonic cultures of the *P. mirabilis* 9B-m strain (62.5–1000 ng ml^−1^) at 37 °C for 48 h. LPS of *E. coli* O55:B5 (Sigma, kindly provided by A. Torzewska, Ph.D., the Department of Biology of Bacteria, University of Łódź) was applied as a positive control of the THP-1 activity. THP-1 macrophages, not stimulated with LPS, were used as a negative control. Next, THP-1 cells were centrifuged, and culture supernatants were collected and frozen at − 20 °C, until analyses. The studies were performed in three independent replicates.

### Assessment of the tumour necrosis factor alpha (TNF-α) level in response to lipopolysaccharide stimulation, statistical analysis

TNF-α concentrations in obtained culture supernatants were determined using the *Ready-Set-Go* ELISA kit (eBioscience) according to the manufacturer’s instructions. Statistical analyses were performed using CCS StatSoft Statistica 12.5 PL. Differences between TNF-α levels in response to LPS stimulation were evaluated using the nonparametric Kruskal–Wallis test (*p* values < 0.05 were considered significant).

### Degradation of the lipopolysaccharide, isolation of lipid A, O-polysaccharide, and core oligosaccharide

Acid degradation of LPS was performed with 1% aqueous acetic acid at 100 °C until precipitation of lipid A (2 h). The precipitate was separated by centrifugation (13,000×*g*, 20 min), and the supernatant was fractionated by gel-exclusion chromatography on a column (56 × 2.6 cm) of Sephadex G-50 (Amersham Biosciences, Sweden) in 0.05 M pyridinium acetate buffer (pH 4.5) monitored using a differential refractometer (Knauer, Germany) to give a high-molecular O-polysaccharide fraction (I) and core oligosaccharide fractions (II): one from planktonic cells (IIa) and two from biofilm-associated cells (IIa and IIb).

### Analyses of O-polysaccharides

Polysaccharide samples (0.5 mg) were hydrolyzed with 2 M trifluoroacetic acid (120 °C, 2 h), acetylated with an acetic anhydride–pyridine mixture (1:1), and analyzed by GLC on an HP-5 ms column (25 m × 0.25 mm) using a Hewlett–Packard 5890 instrument (USA) and a temperature gradient of 160 °C (1 min) to 250 °C at 3 °C min^−1^ or a Biotronik LC-2000 amino acid analyzer as described [29].

NMR spectra of the polysaccharides were recorded with a Bruker DRX-500 spectrometer for solutions in ^2^H_2_O at 30 °C using internal acetone (*δ*_H_ 2.225, *δ*_C_ 31.45) as reference. Standard Bruker software (XWINNMR 2.6) was used to acquire and maintain the NMR data. A mixing time of 200 and 100 ms was applied in two-dimensional TOCSY (total correlation spectroscopy) and ROESY (rotating-frame Overhauser spectroscopy) experiments, respectively.

### Analyses of core oligosaccharides and lipids A

Core oligosaccharides were analyzed by ESI HR MS (electrospray ionization high-resolution mass spectrometry) in the negative mode using a micrOTOF instrument (Bruker Daltonics). Capillary entrance voltage was set to 3200 V, and the drying nitrogen temperature was 180 °C. The samples were dissolved in a 1:1 (v/v) H_2_O/MeCN mixture (∼ 50 ng µl^−1^) and sprayed at a flow rate of 3 µl min^−1^.

Lipid A samples were analyzed by mass spectrometry in the negative reflectron mode using a BRUKER UltrafleXtreme MALDI-TOF MS (matrix-assisted laser desorption/-ionization time-of-flight mass spectrometry) instrument. 9H-Pyrido[3,4-b]indole [10 mg ml^−1^ in a 1:1 acetonitrile/water mixture (v/v)] was used as a matrix. The samples were desalted by extraction with a water/chloroform mixture (1:1, v/v, 1 mg ml^−1^) and dissolved in a methanol/chloroform solution (1:1, v/v, 1 mg ml^−1^). The sample/matrix mixture (1:1 v/v) was deposited (1 µl) onto a stainless steel plate, and left to air dry.

## Results

**Fig. 1:**
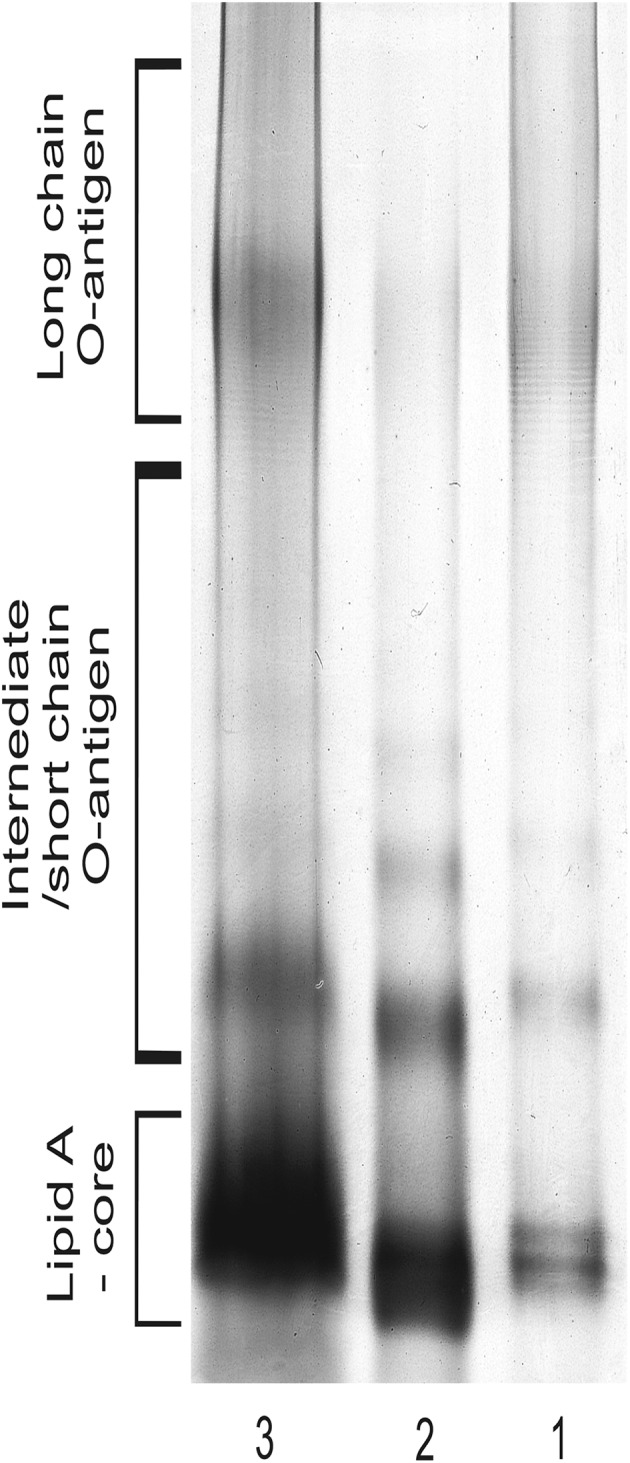
Silver-stained SDS–PAGE electropherogram of the *P. mirabilis* 9B-m lipopolysaccharides: from planktonic (1), biofilm-growing (2) and planktonic, reverted from sessile (3) cells

The present study was designed to investigate changes in the LPS of the *P. mirabilis* 9B-m strain in response to a switch between the planktonic and biofilm form of growth. The LPSs from both types of cultures were obtained (the yields amounted to 6.26 and 5.3% for planktonic and biofilm forms, respectively) and first characterized by SDS–PAGE and silver staining. Figure 1 shows an electropherogram of the LPSs from both (planktonic and biofilm) types of *P. mirabilis* 9B-m cultures. LPS from the planktonic culture displays a pattern with a fast-migrating fraction corresponding with low-molecular mass (O-polysaccharide-lacking) molecules, as well as a slower migrating, high-molecular mass fraction referring to the molecules containing a long-chain O-antigen region. In contrast, LPS derived from sessile cells differs in its electrophoretic pattern by a decrease in the slow-migrating fraction. LPS containing high-molecular-weight OPS was observed again in the cells reverted to the planktonic form.

**Fig. 2:**
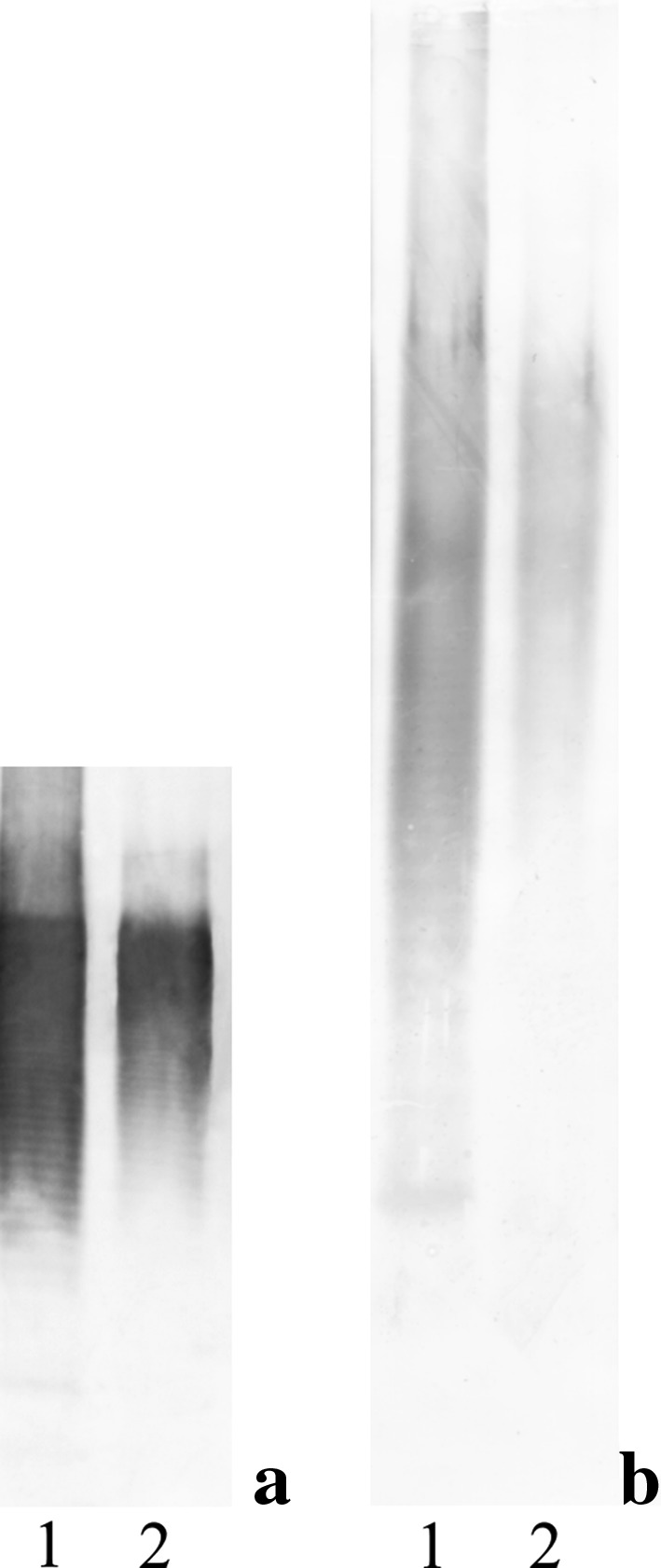
Western blot of LPSs from planktonic (1) and biofilm-associated (2) cells of *P. mirabilis* 9B-m strain with *P. mirabilis* 9B-m O-antiserum: **a** is the short and **b** extended path of separation

We have also checked whether the changes in the lifestyle of *P. mirabilis* 9B-m can affect the profile of serological reactions of LPSs from both types of cultures. To answer this question, serological investigations using the ELISA and Western-blotting techniques were performed. In ELISA, the reactivity of the investigated LPSs was examined with polyclonal O-antiserum against *P. mirabilis* 9B-m (obtained for planktonic forms). To compare serological specificities of the used LPSs, two variants of the serum were applied: non-adsorbed and adsorbed with *P. mirabilis* 9B-m biofilm or planktonic cells. The results of this part of work are presented in Table 1. Antigens from biofilm or planktonic cultures reacted similarly, but non-identically with native O-antiserum (a slightly weaker reaction was observed for LPS from biofilm cells). In addition, the use of the adsorption procedure allowed noting some differences in the reactivity profiles. There was no reactivity observed for both LPSs when the antiserum was adsorbed with planktonic cells (as was mentioned above, O-antiserum had been obtained for planktonic cells). However, when sessile bacteria were added to the native antiserum to remove cross-reacting antibodies, no complete abolition of reactivity with the LPS from planktonic cells was noted. The serum still contained antibodies which reacted with this LPS to the titre 1:4000 (Table 1). These differences in reactivity of both types of LPSs prompted us to use the Western-blotting technique. The results of this stage of investigations are presented in Fig. 2. Qualitative differences in the LPS separation profiles are shown both on the short and on the extended path of electrophoretic separation. The investigated O-antiserum recognized and reacted much stronger with the epitopes in the LPS from planktonic cells. The reaction with the LPS from biofilm cells was much weaker in the fraction of molecules containing the highest molecular-weight O-antigen and, which is particularly noticeable, there was no visible reaction with the fast-migrating LPS fraction (lipid A-core oligosaccharide).

**Table 1:** Reactivity of *P. mirabilis* 9B-m O-antiserum in ELISA with homologous lipopolysaccharides from different types of culture (planktonic or biofilm)

O-antiserum against *P. mirabilis* 9B-m planktonic strain	Reciprocal titre for *P. mirabilis* 9B-m lipopolysaccharide	
	From planktonic culture	From biofilm culture
Non-adsorbed	1,024,000	256,000
Adsorbed with planktonic biomass	< 1000	< 1000
Adsorbed with biofilm biomass	4000	< 1000

The obtained results suggested the form of growth of the investigated *P. mirabilis* clinical strain might influence the changes in the length of polysaccharide chains of the LPS molecules on the bacterial cells, or even the modification of the chemical structure of this component. Therefore, in the next stage of our investigation, chemical analyses were performed.

### Determination of the O-polysaccharide structure

**Fig. 3:**
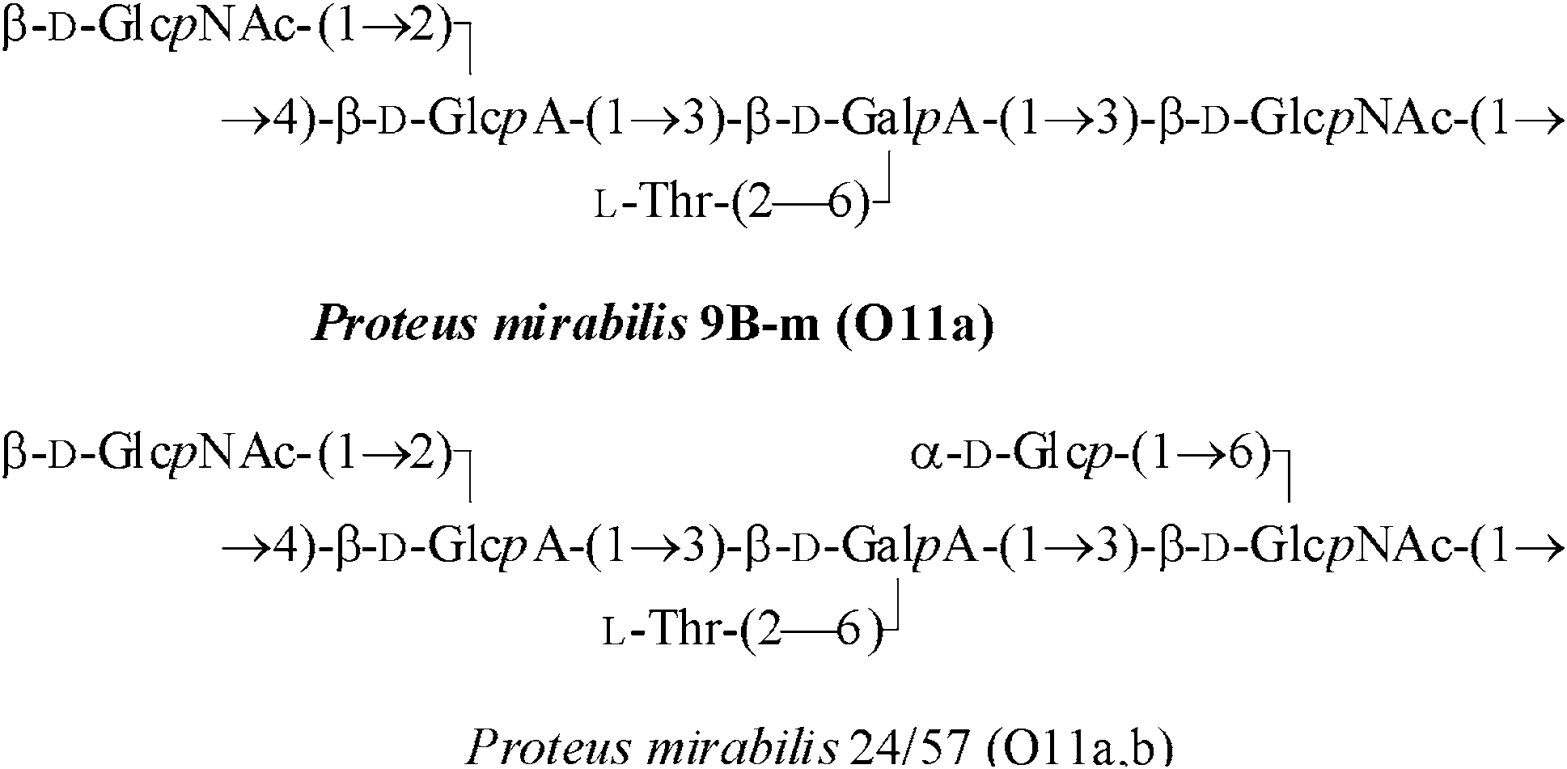
Structures of the related O11-polysaccharides of *P. mirabilis* [30, 31]

Recently, the structure of the OPS of LPS from planktonic *P. mirabilis* 9B-m cells has been elucidated [30]. It has been found that the 9B-m OPS is closely related to that of *P. mirabilis* 24/57 (serogroup O11), which differs by the presence of an additional side-chain Glc residue only [31] (Fig. 3). Therefore, it has been proposed to divide *Proteus* serogroup O11 into two subgroups, including subgroup O11a for the strain 9B-m [30].

Our studies by GLC and NMR spectroscopy showed that the OPS of LPS from biofilm-associated *P. mirabilis* 9B-m cells had the same structure as that from planktonic cells. The ^1^H and ^13^C NMR spectra obtained for the samples from both types of cultures were essentially identical to each other and to those reported earlier [30]. This finding indicates that the outer part of the *P. mirabilis* 9B-m LPS having the structure shown in Fig. 3 did not change under the applied conditions.

### Analyses of the core oligosaccharide

**Fig. 4:**
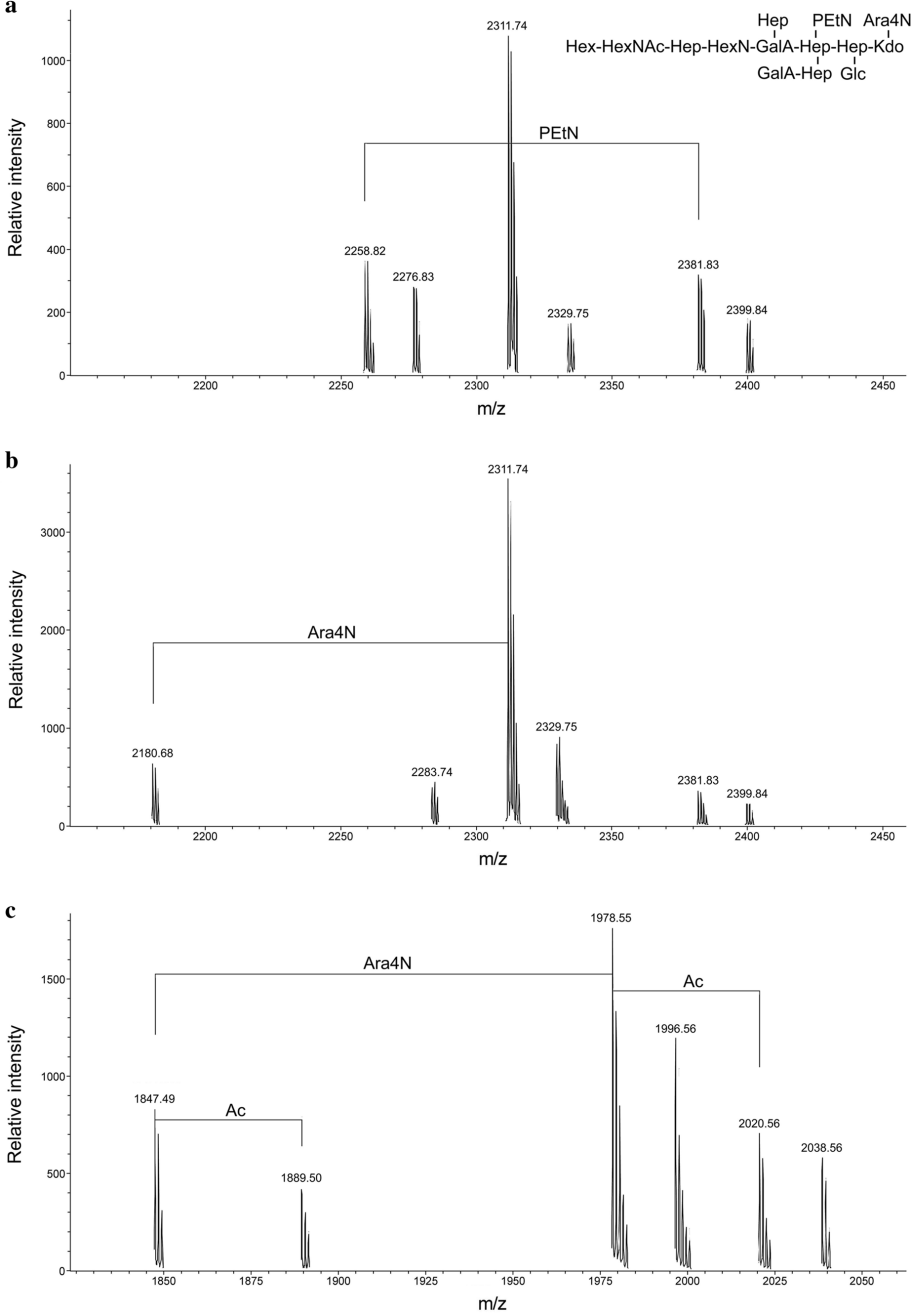
Parts of ESI HR mass spectra of core oligosaccharides from LPS of *P. mirabilis* 9B-m: **a** fraction IIa from planktonic cells; **b, c** fractions IIa and IIb, respectively, from sessile cells. A proposed structure of the fraction IIa major core species corresponding to the [M−H]^−^ ion at *m*/*z* 2311.74 is shown in the inset

Fraction IIa core oligosaccharides were studied by negative ion mode high-resolution electrospray ionization mass spectrometry. The charge deconvoluted mass spectra of the compounds from both kinds of cells (Fig. 4a, b) showed the same major [M−H]^−^ ion peak at *m*/*z* 2311.74, which could be assigned to HexNAc_1_HexN_1_HexA_2_Hex_1_Hep_5_anhKdo_1_Ara4N_1_PEtN_1_ compound, where anhKdo, Ara4N, and PEtN indicate an anhydro form of 3-deoxy-d-*manno*-oct-2-ulosonic acid, 4-amino-4-deoxyarabinose, and phosphoethanolamine, respectively. This and all other peaks in the mass spectra were accompanied by less intense peaks of higher masses by 18 a.m.u. for the corresponding compounds containing a regular Kdo residue. The 9B-m fraction IIa core may represent a minor core species of *P. mirabilis* O34 LPS (Fig. 4, inset), which differs from the major core species by the lack of one of the PEtN groups and a terminal Hex residue [32]. In addition, both spectra contained a minor ion peak at *m*/*z* 2381.82 for an unidentified core oligosaccharide, and the spectrum of the sample from planktonic cells also contained a peak at *m*/*z* 2258.81 for the same oligosaccharide lacking a PEtN group. The spectrum of the sample from the sessile cells showed a minor peak for the compound lacking an Ara4N residue.

The most important difference between core oligosaccharides of LPS from sessile and planktonic cells is presented in Fig. 4c. The mass spectrum of the fraction IIb core oligosaccharide, which was present in the products from biofilm-associated cells only, showed [M−H]^−^ ion peaks at *m*/*z* 1978.54 and 1847.48 for compounds differing by the presence or absence of an Ara4N group, as well as for the corresponding monoacetylated compounds. It needs to be highlighted that these peaks could not be assigned to any known *Proteus* core oligosaccharide [32, 33].

### Analyses of lipid A

**Fig. 5:**
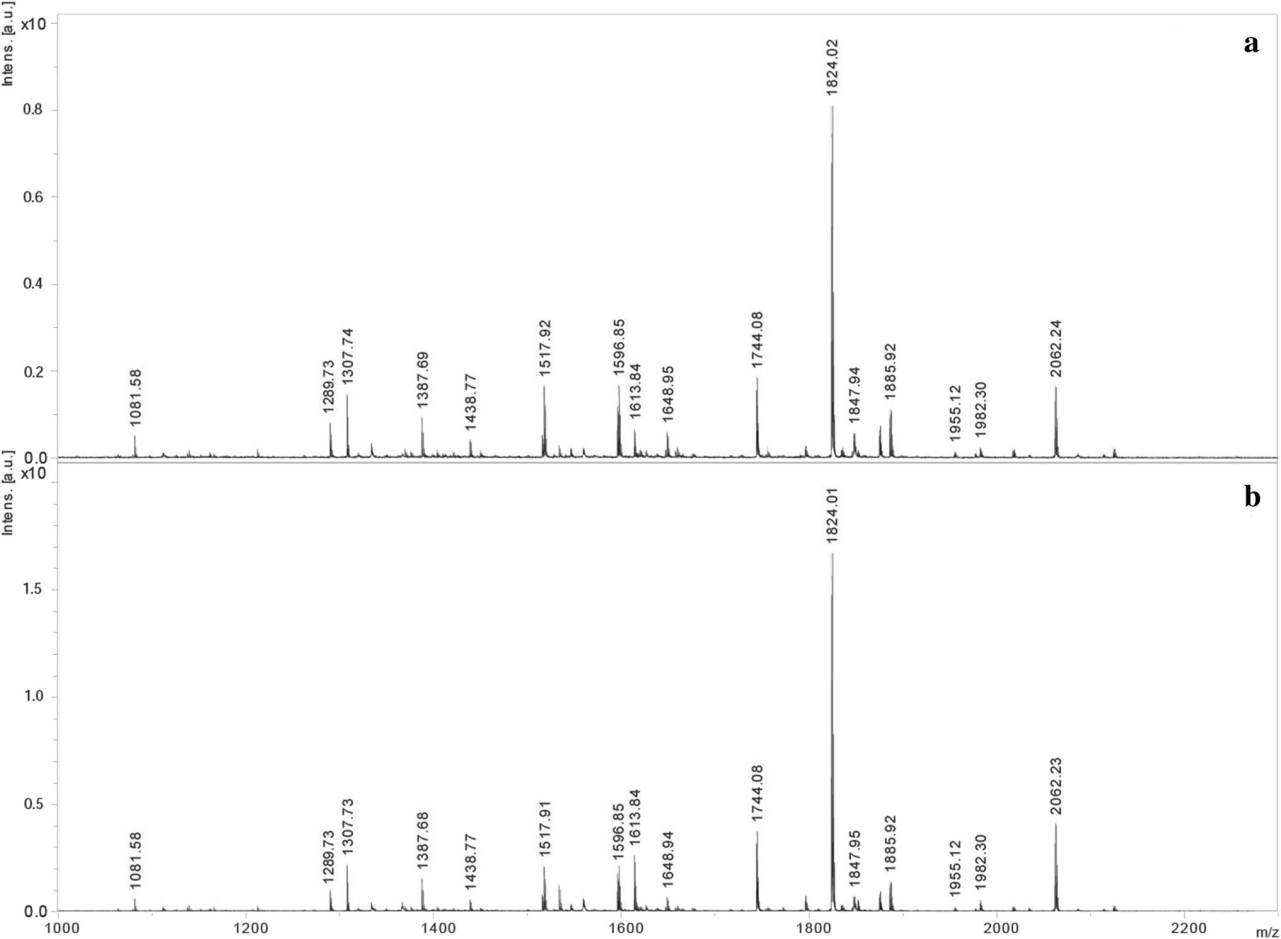
Negative ion mode MALDI-TOF mass spectra of lipid A from LPS of biofilm (**a**) and planktonic (**b**)-associated *P. mirabilis* 9B-m cells

To compare lipid A samples isolated from LPS of planktonic and biofilm-associated *P. mirabilis* 9B-m cells, MALDI-TOF MS analysis was performed. The spectra of lipids A (Fig. 5) showed heterogeneity and had an identical pattern of ions indicating a common structure of both examined lipid A samples. The interpretation of the observed ions was in agreement with previously published data [34]. The most abundant ion at *m*/*z* 1824.02 represented the main population in the *P. mirabilis* 9B-m lipid A, corresponding to the bisphosphorylated and hexaacylated molecule with the calculated mass of two GlcN molecules, two phosphates, four 3-hydroxytetradecanoic [14:0(3-OH)], and two tetradecanoic (14:0) fatty acids. The ion at *m*/*z* 1744.08, which exhibited lower intensity, represented the monophosphorylated form of the main structure. The mass difference 238 Da between *m*/*z* 2062.23 and *m*/*z* 1824.01 indicated the presence of additional hexadecanoic acid (16:0) in lipid A. Additional forms of lipid A were also identified, but the observed ions were detected with lower intensity. Ions at *m*/*z* 1613.84 and 1387.68 could represent different bisphosphorylated molecules devoid of 14:0 or both 14:0 and 14:0(3-OH), respectively. The ion at *m*/*z* 1307.73 could be attributed to a monophosphorylated form devoid of 14:0 and 14:0(3-OH). The ion at *m*/*z* 1517.91 could represent a monophosphorylated species devoid of 14:0(3-OH). Lipid A variants with an additional Ara4N residue were represented by peaks at lower intensities at *m*/*z* 1955.12 (bisphosphorylated form), 1648.94 [monophosphorylated form devoid of 14:0(3-OH)], and 1438.77 [monophosphorylated form devoid of 14:0 and 14:0(3-OH)].

### Analysis of TNF-α secretion

**Fig. 6:**
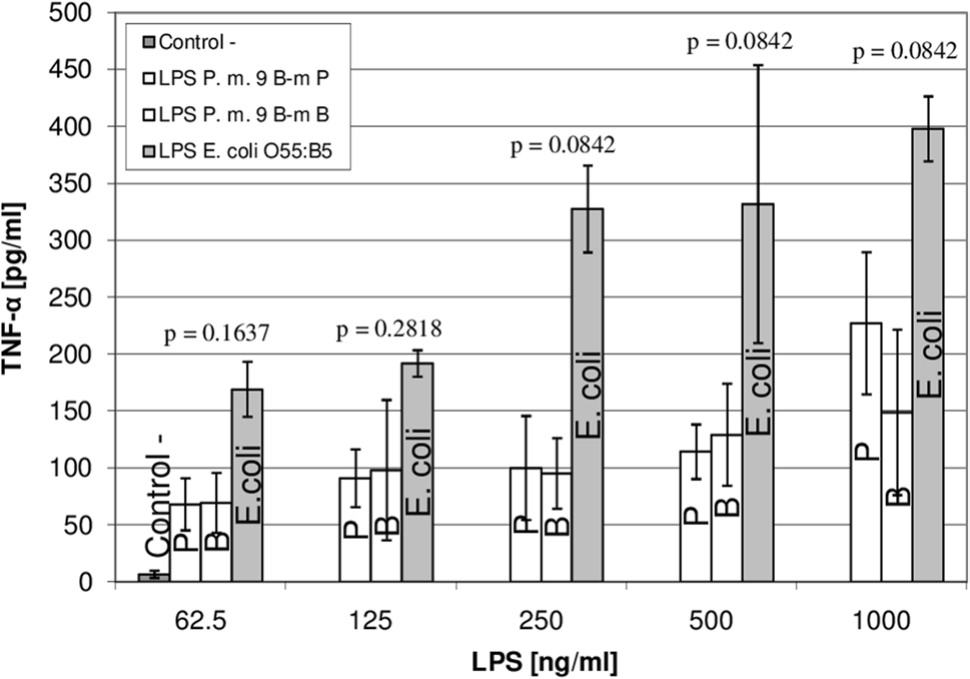
Secretion of TNF-α by THP-1 cells stimulated with *P. mirabilis* 9B-m LPSs from planktonic (P) and biofilm (B) cultures. Data expressed as the median ± SD. P. m., *P. mirabilis*; Control^−^, THP-1 macrophages without LPS; *p*, specific significance values

Since LPS is considered as a classic pro-inflammatory stimulus that leads to TNF- α release, we checked whether the described differences observed in LPSs from both: planktonic and sessile cells influenced the level of TNF-α secretion by THP-1 monocytic cell-line-derived macrophages (results shown in Fig. 6, detailed information under the chart).

LPSs obtained from both planktonic and biofilm-associated *P. mirabilis* 9B-m cells stimulated THP-1 maturated macrophages much more weakly than, used as a positive control, *E. coli* O55:B5 LPS. No significant differences (*p* > 0.05) in the TNF-α concentration were observed between LPSs derived from *P. mirabilis* 9B-m cells cultured as free-floating and sessile microorganisms (irrespective of the used LPS concentration).

## Discussion

It has been demonstrated that many bacteria exhibit different properties while growing in liquid media or on a solid surface as biofilm [35], but little is known about the influence of the growth type on the modification of LPS of Gram-negative microorganisms. Most data refer to *P. aeruginosa* strains and point to the alteration in both OPS and more internal LPS areas [22, 36, 37]. *P. mirabilis* bacilli often form biofilm during urinary tract infection (especially on the intraluminal catheter surface). There is no information how changes of the lifestyle modify the synthesis of LPS of *Proteus* spp. cells. To the best of our knowledge, our report is the first to indicate that this outer membrane heteropolymer may undergo modifications when *Proteus* bacteria mode of life is changed.

We have demonstrated that LPS of the *P. mirabilis* 9B-m clinical strain undergoes some alterations depending on planktonic or biofilm lifestyle. The investigated *P. mirabilis* strain represents the described *Proteus* O11 serotype, which seems to be one of the most frequently isolated from patients [38], also in central Poland [30]. Such high frequency of isolation of *Proteus* spp. strains possessing an O-antigen identical with that of the investigated strain indicates that the chemical structure of OPS of these strains may be beneficial during macroorganism colonization. *P. mirabilis* 9B-m strain intensively forms biofilm—in the group of 30 clinical isolates pre-selected for the study (including 12 clinical *Proteus* O11 isolates, as well as 10 and 8 strains representing O78 and O3 serotypes, respectively), it is one of the three strains form biofilm most efficiently (data not shown). At the same time, the structure of the O-specific part of its LPS was established [30]; thus, this strain was the best candidate for the presented research.

Using the silver-staining technique we showed that in response to changes in the lifestyle, the investigated strain synthesized LPS containing an O-antigen exhibiting a different molecular mass (Fig. 1). In sessile cells, there was observed a reduced number of long-chain O-specific units, whereas in planktonic cells (also those reverted from biofilm), the full-length OPS phenotype was visible. These findings indicate that the observed changes are growth state dependent.

This conclusion was confirmed by the results of Western blotting (Fig. 2), where only a weak reaction was seen within the fraction of the biofilm-associated LPS corresponding to the molecules with a long-chain OPS. The shortening of the LPS polysaccharide chains in sessile cells was also observed in the studies of other opportunistic pathogens like *P. aeruginosa* or food-borne *Cronobacter sakazakii* [22, 36, 37, 39] as well as in the environmental nitrogen-fixing *Bradyrhizobium japonicum* strains [40].

The serological results suggested that the LPS of *P. mirabilis* 9B-m biofilm-living strain could also undergo structural modifications, because changes in its serospecificity were observed. Various reaction titres were found when the native (non-adsorbed) O-antiserum was checked in ELISA with the LPS from planktonic or sessile cells. Using the serum adsorbed by biomass of planktonic cells of the investigated strain showed the lack of reaction with LPSs derived from both planktonic and biofilm cells. Such bilateral abolition of reactivity did not take place when biofilm-forming bacteria were used for adsorption (Table 1). In our previously reported investigations concerning serological classification of *Proteus* spp. strains, we have demonstrated several times that some pool of antibodies remaining in the serum are observed when slight changes in the O-antigen structure or modifications of the LPS core region take place [41–44]. Likewise, in the Western-blotting technique, the differences between the investigated types of LPSs were revealed (Fig. 2). Anti-core antibodies in the O-specific serum (against planktonic *P. mirabilis* 9B-m cells) used in the study have no ability to bind the epitopes located in the prevalent fast-migrating molecules of the LPS from sessile cells. This clearly demonstrate that serospecificity of these epitopes in biofilm-associated LPS is changed that pointed to differences in the chemical structure of LPS in the biofilm in the reference to planktonic mode of growth.

NMR spectroscopy revealed that OPSs from the planktonic and biofilm-associated cells possessed the same structure (Fig. 3); therefore, the form of growth of the *P. mirabilis* 9B-m strain had no effect on the chemistry of its OPS. The obtained results indicate that changes observed in the slow-migrating fraction of biofilm-associated LPS using silver staining (Fig. 1) as well as Western-blotting technique (Fig. 2) were related only to the loss of long-chain OPS, but not to the modification of the chemical structure of this region.

Comparison of the core region structures of LPSs from planktonic and sessile cells revealed changes in the chemistry of that inner part of the *P. mirabilis* 9B-m LPS. We have observed that when bacteria grow in biofilm, the core region in their LPS gains an additional oligosaccharide (ion peaks at *m*/*z* 1978.54 and 1847.48, Fig. 4c). As was mentioned above, none of the currently known core oligosaccharides in the genus *Proteus* was characterized by this kind of peaks. Moreover, this is the first information on the extension of the core part of LPS in bacterial cells forming a biofilm. Most literature data available indicate that truncation of the outer core in LPS is connected with the sessile mode of bacterial growth, and significantly influences biofilm formation. Isogenic mutants of the *P. aeruginosa* PAO1 wild strain, with a truncated outer core, produced LPS lacking the long-chain OPS in biofilm. The study also demonstrated that mutants without OPS showed increased cohesion (cell-to-cell adherence) and adhesion to the glass, which resulted in a more intensive formation of biofilms [45]. This confirmed an earlier observation indicating the loss of polysaccharide chains in LPS of a *P. aeruginosa* biofilm-forming strain [46]. The importance of the modification of the outer core for the intensification of the biofilm formation has also been demonstrated in *Campylobacter jejuni* cells, the species which does not possess the O-antigen in LPS, as well as in series of deep rough mutants of *E. coli*. In the both case, mutants characterized by the significant shortening of the core oligosaccharide formed biofilm more intensively than the parental strain [47, 48]. All the above observations indicate that the modification of the LPS core region during biofilm development resulted from the strain’s increased ability to adhere through the loss of OPS. Although in most cases, the reduction of OPS in LPS was probably due to the truncation of the core region, in the case of the investigated *P. mirabilis* strain, a decrease in the number of full-length O-specific chains attached to the core may have resulted from the emergence of additional oligosaccharide in this region. Further analyses including the determination of the exact structure of the new core oligosaccharide, and the use of a mutant lacking this component would allow elucidating the role played by this additional oligosaccharide during the bacterial biofilm development.

Literature data also show that modification of LPS in biofilm can be related to the lipid A part [22, 49]. In the biofilm formed by *P. aeruginosa*, the changes in lipid A molecules are manifested by increased hydroxylation of the secondary fatty acid chains [22] and in *Escherichia coli* by increased palmitoylation [49]. In the first case, the observed modification led to a slightly higher inflammatory response: TNF-α and interleukin-6 (Il-6) production by human monocytes. Palmitoylation observed in the last case may have enhanced resistance to some antimicrobials and increase survival of bacteria growing in biofilm (in a rat model of catheter infection). MALDI-TOF MS analysis of lipids A derived from planktonic as well as biofilm-associated *P. mirabilis* 9B-m strain showed no differences in the structures of these molecules (Fig. 5). Moreover, the results were confronted with those obtained by Sidorczyk and co-workers [34] for lipid A of *Proteus mirabilis* Re-mutant and two other *P. mirabilis* clinical isolates (data not showed), and again, no alteration was found. This may indicate that in *P. mirabilis* cells, changes in the form of growth do not affect the lipid A synthesis. Interestingly, we have also found no increase in the level of palmitoylation in lipids A under the investigated conditions. The lack of the lipid A modification proved in chemical analyses in the *P. mirabilis* 9B-m planktonic and biofilm-associated strain (and in mentioned two other clinical strains—data not showed) was also reflected by in the results of TNF-α production by THP-1 monocytic cell-line-derived macrophages. In the studies on *P. aeruginosa* strains [22], it was shown that modifications of lipid A occurring during these microorganisms grow in the biofilm and resulted in changes in the TNF-α secretion. In contrast, in our study, no statistically significant differences were observed in the level of this cytokine produced in response to the presence of LPS from both growth states of the *P. mirabilis* 9B-m isolate (as well as of the two other clinical *P. mirabilis* strains mentioned above—data not shown).

In conclusion, we have shown that in *P. mirabilis*, the changes of the form of cells growth may induce alterations in the LPS synthesis. These modifications may affect the most external part of LPS (O-specific part), and the core region but do not concern lipid A part.
